# Preliminary Study on the Use of Chitosan as an Eco-Friendly Alternative to Control *Fusarium* Growth and Mycotoxin Production on Maize and Wheat

**DOI:** 10.3390/pathogens8010029

**Published:** 2019-03-05

**Authors:** Vanessa G. L. Zachetti, Eugenia Cendoya, María J. Nichea, Sofía N. Chulze, María L. Ramirez

**Affiliations:** 1 Departamento de Microbiología e Inmunología, Facultad de Ciencias Exactas Fco-Qcas y Naturales, Universidad Nacional de Río Cuarto. Ruta 36 Km 601, (5800) Río Cuarto, Córdoba X5804BYA, Argentina; vzachetti@exa.unrc.edu.ar (V.G.L.Z.); ecendoya@exa.unrc.edu.ar (E.C.); mnichea@exa.unrc.edu.ar (M.J.N.); schulze@exa.unrc.edu.ar (S.N.C.); 2 Consejo Nacional de Investigaciones Científicas y Técnicas, CONICET, Godoy Cruz 2290, CABA, Buenos Aires C1425FQB, Argentina

**Keywords:** chitosan, *Fusarium*, fumonisin, deoxynivalenol, wheat, maize

## Abstract

The objectives of the present study were to determine the combined effects of chitosan and water activity (a_W_) on growth and mycotoxin production in situ on the two most important *Fusarium* species (*F. proliferatum* and *F. verticillioides*) present on maize, and on *F. graminearum*, the main pathogen causing Fusarium head blight on wheat. Results showed that low-molecular-weight chitosan with more than 70% deacetylation at the lowest dose used (0.5 mg/g) was able to reduce deoxynivalenol (DON) and fumonisin (FBs) production on irradiated maize and wheat grains. Growth rates of *F. graminearum* also decreased at the lowest chitosan dose used (0.5 mg/g), while *F. verticillioides* and *F. proliferatum* growth rates were reduced at 0.98 a_W_ at the highest chitosan dose used (2 mg/g). Since mycotoxins are unavoidable contaminants in food and feed chains, their presence needs to be reduced in order to minimize their effects on human and animal health and to diminish the annual market loss through rejected maize and wheat; in this scenario, pre- and post-harvest use of chitosan could be an important alternative.

## 1. Introduction

*Fusarium* is one of the most economically important genera of phytopathogenic fungi. Several *Fusarium* species can infect small grain cereals (wheat, barley, and oat) and maize causing losses by seedling blight or reducing seed germination, or by causing seedling foot and stalk rots. However, the most important diseases in cereals, due to a severe reduction in yield and quality, are head blight of small cereals such as wheat, and ear rot of maize. Also, another risk is the presence of *Fusarium* toxins that contaminate cereals and are of concern because they could result in harmful contamination of foods and feedstuffs [1]. Maize can be infected by many toxigenic fungi [1,2,3], including *Fusarium verticillioides* and *F. proliferatum* [3,4,5,6,7]. These *Fusarium* species belong to the *Fusarium fujikuroi* species complex (FFSC) and are important pathogens of maize that can produce a high number of mycotoxins. Among them, fumonisins (FBs) are the most important in terms of occurrence and levels. Fumonisins are polyketides which are structurally similar to cellular sphingolipids, and they were shown to inhibit sphingolipid biosynthesis via the ceramide synthase pathway. Fumonisin toxicity is thought to result from the blockage of sphingolipid biosynthesis [8]. Dietary exposure to these mycotoxins was associated with leukoencephalomalacia in equine, hepatic, and renal toxicity in rodents, pulmonary edema in pigs, and esophageal cancer and neural tube defects in humans [9,10,11,12,13]. Recent studies suggest that exposure to fumonisins could also be related to stunting in children [14,15]. Due to the toxicological effect in humans and animals, fumonisin B_1_ (FB_1_) is classified as a “2B” carcinogen by the International Agency of Research on Cancer [16]. The Joint Food and Agriculture Organization and World Health Organization (FAO/WHO) Expert Committee on Food Additives (JECFA) determined a provisional maximum tolerable daily intake (PMTDI) of 2 µg/kg body weight per day for FB_1_, fumonisin B_2_ (FB_2_), and fumonisin B_3_ (FB_3_) alone or in combination [17]. Maximum fumonisin limits for human consumption in cereals and cereal-based foods were established by the European Union in 2007 (EC N°1126/2007), being 1000 ng/g for maize and sub-products used for human consumption.

One of the most important diseases of wheat and other cereals in many areas of the world is Fusarium head blight (FHB). This disease causes yield losses and often results in the accumulation of *Fusarium* mycotoxins in wheat grains [18]. In Argentina, the main pathogen associated with this disease is *Fusarium graminearum sensu stricto* [19,20,21]. Consequently, wheat is often contaminated with mycotoxins, with deoxinivalenol (DON) as the predominant one [19,22,23,24]. DON belongs to the type B trichothecenes. Historically, DON, also called vomitoxin, was notorious since it causes acute and chronic disease symptoms in humans and animals that consume contaminated grains [25]. Its toxic effects range from diarrhea to vomiting, gastro-intestinal inflammation, and necrosis of the intestinal tract, the bone marrow, and the lymphoid tissues [26]. Regarding DON legislation, the European Union sets limits of 1250 ng/g for unprocessed cereals, other than durum wheat, oats, and maize, and 1750 ng/g for unprocessed durum wheat and oats, while the United States of America (USA) and Canada legislation sets 2000 and 1000 ng/g, respectively, in wheat and wheat sub-products used for human consumption. 

Considering the high impact of FBs and DON on human and animal health, and the economic losses related with the contamination of maize and wheat grains with mycotoxins, it is important to develop strategies in order to prevent their formation and/or to eliminate, inactivate, or reduce their presence in both cereals and food products. For this, several strategies such as a combination of agronomical practices, resistant cultivars, and fungicides are used [27]. However, chemical fungicides are reported to cause problems such as ambient pollution, chemical residues in food and feed, and the development of resistant pathogenic fungi [28]. Also, under certain conditions, they may act as a stress factor resulting in the induction of toxin biosynthesis; sub-lethal doses of some fungicides may lead to a stimulation of mycotoxin production by *Fusarium* species [29,30]. Due to these problems, there is an increased interest in the study and use of antifungal compounds obtained from natural sources to replace synthetic fungicides. Chitosan emerged as a promising eco-friendly alternative since it can be used to produce biodegradable fungicides. It induces several biological responses in plants; it enhances defense responses to abiotic and biotic stresses, stimulates plant growth, stimulates the effect of different enzyme activities to detoxify reactive oxygen species, interacts with chromatin, and directly affects gene expression [31]. Also, it is used to protect seeds [32]. Most importantly, it is considered as generally recognized as safe (GRAS) by the Food and Drug Administration (FDA). Chitosan is a deacetylated form of chitin and has the ability to inhibit a wide variety of microorganisms such as fungi, bacteria, and viruses [33,34]. Chitosan is a lineal copolymer of β-(1,4) 2-acetamido-2deoxy-β-d-glucopyranose and 2-amino-2-deoxy-β-d-glucopyranose [35], and its biological properties are attributed to several traits, including deacetylation degree and molecular mass concentration [36]. Also, recently, the use of chitosan as a food preservative or adjuvant in agriculture increased to protect or stimulate the defense of different crops [33,34]. 

To date, only a few studies reported the effect of chitosan on both growth and mycotoxin production in different fungal species. For instance, on *Aspergillus flavus* and *Aspergillus parasiticus* [37,38,39] and *Alternaria alternata* f. sp. *lycopersici* [40]. It is surprising that these reports took no account of the interactions between the efficacy of chitosan and key environmental factors, such as water activity (a_W_) or temperature, since they are known for being main factors that influence fungal growth and mycotoxin production [41].

In a previous study in vitro, we demonstrated the effect of chitosan on growth of *F. verticillioides* and *F. proliferatum* strains, as well as on FB production, under different a_W_ at 25 °C, using a maize-based media. We observed that chitosan was able to significantly reduce growth rate and FB production, with maximum levels of reduction in both parameters obtained at the highest doses used [42]. Ramirez et al. [30] suggested that the effect of antifungal compounds on artificial substrate may not accurately represent the real situation on a natural substrate. The use of irradiated grains that retained viability may be a more appropriate system for screening antifungal and anti-mycotoxin compounds.

Under this scenario, chitosan could be proposed as a possible option to control fungal growth and mycotoxin accumulation in cereals, mainly due to its biological properties and its easy production by partial alkaline *N*-deacetylation of chitin [43]. 

The objectives of the present study were to determine the effect of chitosan under different a_W_ at 25 °C on the (i) lag phase, (ii) growth rate, and (iii) mycotoxin (fumonisin and dexynivalenol) production by *F. verticillioides*, *F. proliferatum*, and *F. graminearum* on irradiated maize and wheat grains.

## 2. Results

### 2.1. Chitosan Characterization

The average chitosan molecular weight (Mw) in g/mol determined by the Mark–Houwink–Sakurada equation was 3.42 ± 0.08 × 10^3^ g/mol. The deacetylation degree (DD), determined by an infrared spectroscopy analysis, was 77.6%.

### 2.2. Effect of Chitosan Concentration and a_W_ on Growth Rates

**Figure 1:**
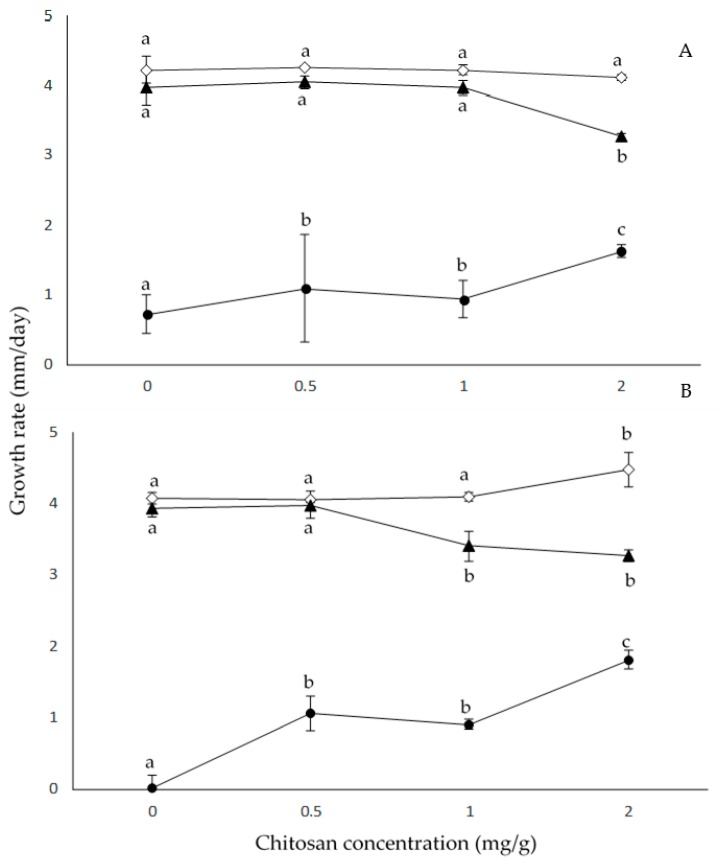
Effect of chitosan and water activity (a_W_) (0.99 (◇), 0.98 (▲), 0.95 (⬤)) on growth rates of *Fusarium proliferatum* RC2080 (A) and *F. verticillioides* M7075 (B) strains on irradiated maize grains. Mean values based on biological triplicate data with letters in common for each a_W_ are not significantly different according to the Tukey test (*p* > 0.001).

Figure 1 summarizes the effect of a_W_ and chitosan doses on *F. proliferatum* RC2080 and *F. verticillioides* M7075 growth rates on irradiated maize grains. The results showed that increasing doses of chitosan at 0.99 a_W_ in both *Fusarium* species did not affect their growth rates, while at 0.98 a_W_ and 2 mg/mL chitosan, growth rates were significantly lower for both *Fusarium* species. Significant increases (*p* > 0.001) in growth rates were observed when *F. proliferatum* RC2080 and *F. verticillioides* M7075 were treated with chitosan at 0.95 a_W_; the increases were around 125% and 181% for *F. proliferatum* RC2080 and *F. verticillioides* M7075, respectively. No statistical differences were observed (*p* > 0.001) when *F. proliferatum* RC2080 was treated with chitosan doses of 0.5 and 1 mg/mL and 0.99 and 0.98 a_W_, regarding the control treatment. *F. verticillioides* M7075 treated with chitosan doses of 0.5 and 1 mg/mL and 0.99 a_W_ showed no statistical differences (*p* > 0.001) compared to the control. 

The statistical analysis using ANOVA of single factors (strain, chitosan dose, and a_W_) and of all interactions (two and three-way) on growth rate showed that the major effect was due to a_W_ followed by the interaction chitosan × a_W_ dose. The strain factor did not significantly affect the growth rate; therefore, it leads us to conclude that the strain behavior was the same in every condition tested (Table 1).

**Figure 2:**
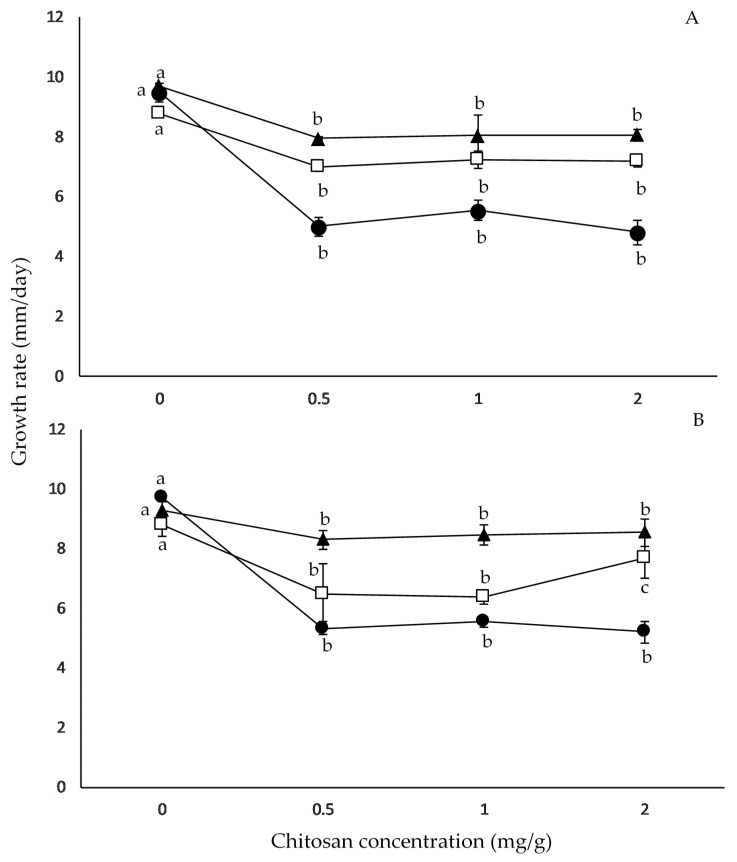
Effect of chitosan and a_W_ (0.995 (▲), 0.99 (⬜), 0.98 (⬤)) on growth rates of *F. graminearum* RCFG6001 (A) and *F. graminearum* RC22-2 (B) strains on irradiated wheat grains. Mean values based on biological triplicate data with letters in common for each a_W_ are not significantly different according to the Tukey test (*p* > 0.001).

Figure 2 summarizes the effect of a_W_ and chitosan doses on *F. graminearum* strain growth rates on irradiated wheat grains. Under control conditions, maximum growth rates were observed at 0.995 and 0.98 a_W_ depending on the *F. graminearum* strain tested. Growth rates of both *F. graminearum* strains significantly decreased when chitosan was used, as compared to the control conditions (Figure 2). The highest growth reduction with chitosan was observed at 0.98 a_W_ for both strains. However, no significant differences were observed among chitosan concentrations at the same a_W_ for both strains. 

The statistical analysis using ANOVA of single factors (strains, a_W_, and chitosan dose) and their interactions on growth rates showed that a_W_ and chitosan dose alone, as well as chitosan × a_W_ interaction, were significant (*p* < 0.001), whereby chitosan was the factor that had the greatest effect on growth rates of both *F. graminearum* strains (Table 2).

### 2.3. Effect of Chitosan Concentration and a_W_ on Mycotoxin Production

The *F. verticillioides* strain was able to produce higher amounts of fumonisins in almost all tested conditions in comparison with *F. proliferatum*. Maximum fumonisin production in the control condition for both strains was observed at 0.98 a_W_, and, at that a_W_, a significant reduction in FB levels was observed when chitosan treatments were used, reaching 88% and 95% reduction for *F. proliferatum* RC2080 and *F. verticillioides* M7075, respectively. For both strains, the lowest amounts of FBs were produced at 0.95 a_W_ in the control condition. A high stimulation in fumonisin production by *F. verticillioides* M7075 was observed when the chitosan dose was 1 mg/g at 0.99 a_W_ (Table 3); for the same strain, no significant differences were observed at 0.95 a_W_ when chitosan was applied, while, for *F. proliferatum*, when chitosan dose was ≥1 mg/g at the same a_W_, FB production was significantly reduced. For both strains, in all conditions, FB_1_ was found in higher amounts than FB_2_ and FB_3_ (data not shown). The statistical analysis using ANOVA showed that single factors (strains, a_W_, and chitosan dose) and some interactions (S × a_W_ and C × a_W_) were significant on fumonisin production. The major effect was given by a_W_, followed by strain factor (Table 4). 

Regarding DON production, maximum concentrations were produced by the two *F. graminearum* strains at 0.995 a_W_ in the control condition (without chitosan addition) on irradiated wheat grains. When chitosan treatments were applied to the irradiated wheat grains, DON levels were lower in comparison with the control condition, with the exception of *F. graminearum* RC22-2 at 0.99 a_W_, where a stimulation in DON production was observed. Overall, it was observed that the *F. graminearum* RC22-2 strain produced higher amounts of DON in comparison with the *F. graminearum* RCFG6001 strain (Table 5). 

DON production by *F. graminearum* RCFG6001 strain was reduced as long as increasing chitosan doses were used, with reductions between 93% and 100% in all treatments and a_W_ tested. DON production by *F. graminearum* RC22-2 was reduced at 0.995 and 0.98 a_W_ when chitosan was applied at all used doses, with the exception of 0.98 a_W_ when 1 mg/g was used and a slight stimulation in DON production was observed. Nevertheless, it was not significant (Table 5).

The analysis of the effect of individual factors (strains, chitosan dose, and a_W_) and most interactions (two- and three-way) on DON production showed that all the factors and interactions were statistically significant. Strain factor was the variable that most affected DON production, followed by chitosan dose and a_W_ (Table 4).

## 3. Discussion

The present study highlights the potential of chitosan for controlling both growth and mycotoxin production (FBs and DON) by important *Fusarium* species, which are common cereal contaminants. However, it is important to consider that different responses to chitosan use were found among different fungal strains. Notably, at the lowest chitosan concentration used, we observed reductions in growth rates and an important effect on DON production by both *F. graminearum* strains, independently of the a_W_. The only exception was observed at 0.99 a_W_ for strain RC22-2. 

Regarding the *Fusarium* species that produce fumonisins (*F. verticillioides* and *F. prolifeartum*), we only observed a significant reduction in growth rates at 0.98 aw for both species at the highest chitosan dose used. Overall, important FB reductions were found at the lowest doses used (0.5 mg/g) at 0.98 a_W_, which is considered the most suitable a_W_ for this mycotoxin production on maize [44]. Moreover, for the other a_W_, the most effective chitosan dose for fumonisin reduction was 1 mg/g with the exception of *F. verticillioides* at 0.99 a_W_.

The antifungal activity of chitosan over a broad range of phytopathgenic fungi was the subject of several review papers [35,45,46,47]. To the best of our knowledge, none of the previous studies reported on the interactions between the efficacy of chitosan or chitosan derivates and a_W_ or temperature, which were demonstrated to be key parameters determining germination, growth, and mycotoxin production by different fungal species [20,48].

Also, there are no other studies in the literature evaluating both the chitosan and a_W_ effect on *Fusarium* species growth and mycotoxin production on irradiated grains. There was only one study in vitro previously carried out in our laboratory by Ferrochio et al. [42], who demonstrated that different chitosan doses from 0.5 to 3.0 mg/L, combined with a_W_ reduction, increased lag phases and decreased mycelial growth rates in *F. verticillioides* and *F. proliferatum* strains, when the study was performed in a wheat-based media at 25 °C. The lowest growth rate was obtained at the highest chitosan concentration tested (3 mg/mL) and at the lowest a_W_ (0.93). However, these results are different from those obtained in the present study using the same *F. verticillioides* and *F. proliferatum* strains on irradiated maize grains. This could be explained by the fact that, although irradiated grains contained all the nutritional elements in order to allow the fungal development, they could represent a natural barrier in the penetration of *Fusarium*, thus restricting the fungal growth. However, good levels of fumonisin reduction were achieved in both studies at the lowest chitosan doses used (0.5 mg/g).

The antifungal activities of chitosan relied on several intrinsic and extrinsic factors, such as pH, fungal species, presence or absence of metal cations, pKa, Mw, DD, etc. [35]. Verlee et al. [45] criticized that most papers dealing with antifungal activity did not report the characterization (Mw and DD) of chitosan. For all these reasons, during the present work, some characteristics of the commercial chitosan used were determined. The results showed that chitosan presents a low molecular weight and 71% DD. For soluble chitosan, pH is a crucial factor related to solubility, and can further alter antifungal activity. The antifungal activity of chitosan is exhibited when the pH is below the representative pKa (~6.5), the value at which the soluble molecule could be dissociated as ions. In the present study, the pH of the chitosan solution was maintained below the pKa.

There were several attempts, in the last few years, to use chitosan for reducing FHB and DON contamination. Khan et al. [49] showed that both chitosan and *Pseudomonas fluorescens* (biological control agent) were very effective in reducing FHB disease caused by *F. culmorum* and DON contamination in wheat and barley grains, when they were applied at high disease pressure under glasshouse and field experiments. They concluded that, overall, chitosan was more effective than the potential biocontrol bacterium *P. fluorescens* strain MKB158. Also, Kheiri et al. [50], reported a good in vitro effect on growth of *F. graminearum* and FHB severity reduction in a glasshouse experiment using chitosan and chitosan nanoparticles, but they did not evaluate DON contamination.

In our group, we demonstrated the efficacy of *Bacillus velezensis* RC218 and *Streptomyces albidoflavus* RC87B for reducing FHB severity and DON accumulation by *F. graminearum* under glasshouse and field experiments [24]. Taking into account the good results obtained during the present work with chitosan and *F. graminearum* in situ, we are developing further experiments combining the biological control agent with chitosan in order to improve the achieved results.

In conclusion, the present study showed the combined effects of chitosan and a_W_ on growth and mycotoxin production by the three most important *Fusarium* species present on maize and wheat. Also, it was demonstrated that low-Mw chitosan with more than 70% DD at 0.5 mg/mL was able to significantly reduce growth rate and mycotoxin production on irradiated grains. It is important to remark that the effect was strain-specific, and, for this reason, these types of antifungal tests need to be made with at least two strains. Since mycotoxins are unavoidable contaminants in food and feed chains, their presence needs to be reduced in order to reduce their effects on human and animal health and to decrease the annual market losses caused by rejected cereals. In this scenario, pre/post-harvest (for *F. graminearum*) and post-harvest (for *F. verticillioides* and *F. proliferatum*) use of chitosan could be an important alternative. The use of chitosan is proposed as a post-harvest alternative treatment in maize in Argentina since permanent storage capacity is not increasing at the same rate as cereal production. Thus, a substantial portion of the harvest (17 million tons) is stored in temporary hermetic storage systems called silo-bags that remain in the field for long periods (over five months). Normally, these bags are filled with maize at 14% to 16% moisture content (wet basis) (0.72–0.8 aw) [51]. Pacin et al. [52] evaluated fumonisin contamination in maize stored in silo-bags and observed that, in maize stored for around 200 days following good agricultural practices, fumonisin contamination increased significantly. Regarding wheat grains, we propose the use of chitosan as a pre- and post-harvest strategy. As the global wheat production increased steadily in recent years, a substantial portion of the harvest is stored under natural conditions with no controlled facilities in many developed countries. Yuan et al. [53] observed that DON levels increased up to 30% after three months of storage. Thus, post-harvest control strategies are necessary, and chitosan could also be used. However, it is necessary to continue with further basic studies that could contribute to explaining the effect of applying this polymer on cereals.

## 4. Materials and Methods

### 4.1. Chitosan Solution

Low-viscosity chitosan was used (Fluka 50494; LVC; viscosity: ≤200 mPa∙s). A stock solution was prepared by dissolving 10 g/L of chitosan in 1% acetic acid (AcH), and the solution was stirred for 24 hours at 28 °C. The pH of the chitosan solution was adjusted to 5.6 using NaOH in order to ensure that all the chitosan amino groups were positively charged [35,54]. Then, the solution obtained was autoclaved at 121 °C for 15 min, and maintained at 4 °C until use.

### 4.2. Chitosan Characterization

The viscosity–average molecular weight (Mw) of chitosan was determined using the intrinsic viscometric method using the Mark–Houwink–Sakurada equation [55]. The percentage of the chitosan amino groups (degree of deacetylation, DD) was determined using an infrared spectroscopy (Bruker Tensor 27) analysis, applying the following equation:

DD (%) = 97.67 − 26.486 × (A_1655_/A_3450_), (1)

where A_1655_ is the absorbance at 1655 cm^−1^ of the amide I band and A_3450_ is the absorbance at 3450 cm^−1^ of the hydroxyl band [37].

### 4.3. Fungal Strains

One *F. verticillioides* (M7075) strain and one *F. proliferatum* (RC2080) strain were used. Both strains were isolated from maize in Argentina [56,57] and were characterized using a polyphasic approach: morphological, biological, and genetic (amplified fragment length polymorphisms; AFLP). Also, fumonisin production capability was demonstrated. Moreover, two strains identified as *F. graminearum sensu stricto*, RC22-2 and RCFG6001, respectively, isolated from wheat spikes with Fusarium head blight (FBH) symptoms, were included in the present study. These strains were identified morphologically, by AFLP, and by partial sequencing of elongation factor gene (EF-1α); DON production capability was also demonstrated in previous studies [21,58]. The strains were deposited at the Department of Microbiology and Immunology, Universidad Nacional de Rio Cuarto culture collection (RC). Cultures were maintained in 15% glycerol at −80 °C (Table S1 in the Supplementary Materials).

### 4.4. Grains

Maize and wheat grains were gamma-irradiated (10–12 kGy) using a cobalt radiation source and were stored aseptically at 4 °C. Grains contained no fungal infection or contamination, but retained germinative capacity. The initial values of a_W_ of the grains were 0.75 and 0.65 for maize and wheat, respectively. Irradiated maize and wheat grains were weighed and placed into sterile flasks and rehydrated to the required a_W_ (0.99, 0.98, and 0.95 for maize, and 0.995, 0.99, and 0.98 for wheat) via addition of sterile distilled water using a moisture absorption curve. An appropriate aliquot of water was replaced by chitosan stock solution to give to the irradiated grains a final concentration (0.5, 1, and 2 mg/g). Flasks were subsequently refrigerated at 4 °C for 48 h with periodic shaking to allow absorption and equilibration. At the end of this period, the a_W_ was checked with an Aqualab Series 3 (Decagon Devices, Inc, WA, USA). The rehydrated maize and wheat were placed in sterile 9-cm Petri dishes to form thin layers of grains (~20 g).

In order to discard fungal inhibition due to acetic acid in the chitosan solution, irradiated grains without chitosan (control AcH) were prepared for each a_W_ level used. Those AcH control plates were prepared by adding the same volume of 1% AcH (pH adjusted to 5.6) used to prepare 2 mg/g of chitosan-amended wheat and maize grains. 

### 4.5. Inoculation, Incubation, and Growth Assessment

All plates were inoculated with a 4-mm-diameter agar disc that was taken from the margin of a seven-day-old colony of each strain grown on synthetic nutrient agar at 25 °C [59]. The discs were transferred face-down onto the center of each plate. Inoculated Petri plates containing grains of the same a_W_ were enclosed in plastic containers together with two beakers of NaCl–water solutions of the same a_W_ as the treatments to maintain constant equilibrium relative humidity (ERH) inside the boxes. Containers were incubated at 25 °C for 28 and 21 days for irradiated maize grains and irradiated wheat grains, respectively. 

Experiments were carried out in triplicate for each treatment. Assessment of growth was made daily during the incubation period, and two diameters of the growing colonies were measured at right angles to each other until the colony reached the edge of the plate. The radii of the colonies were plotted against time, and linear regression was applied in order to obtain the growth rate (mm/day) as the slope of the line. After the incubation period, three complete Petri plate cultures per treatment were sampled, dried at 50 °C for 24 h, and stored at −20 °C until toxin analysis (FB extraction from irradiated maize grains, and DON extraction from irradiated wheat grains).

### 4.6. Mycotoxin Determination

#### 4.6.1. Fumonisin Determination in Maize

Each sample was finely ground and mixed well. A sub-sample (15 g) was extracted with 40 mL of a mixture of acetonitrile–water (1:1 *v*/*v*) for 30 min using an orbital shaker (150 rpm) and then filtering the extracts through a paper filter (Nº 4, Whatman International Ltd, Maidstone, Kent, United Kingdom). An aliquot of extract (1000 μL) was taken and stored in an Eppendorf tube at −20 °C until HPLC analysis. 

An aliquot (50 μL) of this solution was derivatized with 200 μL of an *o*-phthaldialdehyde (OPA) solution obtained by adding 5 mL of 0.1 M sodium tetraborate and 50 µL of 2-mercaptoethanol to 1 mL of methanol containing 40 mg of OPA [60]. The fumonisin OPA derivates (50 μL solution) were analyzed using a reversed-phase HPLC/fluorescence detection system. The HPLC system consisted of a Hewlett-Packard 1100 pump (Hewlett-Packard, Palo Alto, CA, USA) connected to a Hewlett-Packard 1046A programmable fluorescence detector and a data module Hewlett-Packard Kayak XA (HP ChemStation Rev. A.06.01). Chromatographic separations were performed on a stainless-steel, C_18_ reversed-phase column (150 × 4.6 mm inner diameter (i.d.), 5 μm particle size; Luna-Phenomenex, Torrance, CA, USA) connected to Security Guard cartridge (4 × 3 mm i.d., 5 μm particle size; Phenomenex, Torrance, CA, USA) filled with the same phase. Methanol–0.1 M sodium dihydrogen phosphate (75:25, *v*/*v*) solution adjusted to pH 3.35 with orthophosphoric acid was used as the mobile phase, at a flow rate of 1.5 mL/min. Fluorescence of the fumonisin OPA derivatives was recorded at excitation and emission wavelengths of 335 and 440 nm, respectively. Fumonisins were measured as peak heights and compared with reference standard solutions (Sigma Chemical Co, St. Louis, MO, USA). A mixed acetonitrile–water (1:1, *v*/*v*) stock solution of FB_1_, FB_2_, and FB_3_ containing 50 μg/mL of each toxin was prepared. Four mixed working calibrant solutions (0.25, 0.5, 1.0, and 2.0 μg/mL) were prepared by diluting an aliquot of the stock solution with the appropriate volume of acetonitrile–water (1:1, *v*/*v*). The retention times of FB_1_, FB_3_, and FB_2_ were 7.5, 16.7, and 18.5 min, respectively. Appropriate dilutions of standards and/or sample extracts were made with acetonitrile–water (1:1). The detection limit (LOD) of the analytical method for the three fumonisins was 1 µg/g based on the signal-to-noise ratio 3:1. 

#### 4.6.2. Deoxynivalenol Determination in Wheat 

The DON analysis was done using a modified version of that originally reported by Cooney et al. [61]. Each sample was finely ground and mixed well. A sub-sample (15 g) was extracted by mixing with acetonitrile–methanol (14:1; 40 mL), shaken for 2 h, and then filtered through filter paper (Whatman N° 1, Whatman International Ltd, Maidstone, Kent, United Kingdom). A syringe was plugged with glass wool and dry-packed with alumina/carbon (20:1; 300 mg) to form a mini-cleanup column. A 2-mL aliquot of extract was applied to the column and allowed to drain under gravity, and the eluent was collected. The column was washed with 500 µL of a mixture of acetonitrile–methanol–water (80:5:15 *v*/*v*), and the combined eluant was evaporated to dryness using N_2_ at 50 °C. The cleaned-up residue was dissolved in 500 µL of methanol–water (5:95 *v*/*v*) and stored until HPLC analysis. The HPLC system consisted of a Hewlett-Packard model 1100 pump (Palo Alto, CA) connected to a Hewlett-Packard 1100 Series variable wavelength detector and a data module Hewlett-Packard Kayak XA (HP ChemStation Rev. A.06.01). Chromatographic separations were performed on a Luna™ C_18_ reversed-phase column (100 × 4.6 mm, 5 μm particle size) connected to a guard column, SecurityGuard™ (4 × 3.0 mm), filled with the same phase. The mobile phase consisted of methanol–water (12:88, *v*/*v*), at a flow rate of 1.5 mL∙min^−1^. The detector was set at 220 nm with an attenuation of 0.01 AUFS (absorbance units full scale). The injection volume was 50 μL. Quantification was relative to external standards of DON (Sigma-Aldrich Co. St Louis, MO) of 1 to 4 μg/mL in methanol–water (5:95). The quantification (LOD) limit was 5 ng/g.

### 4.7. Statistical Treatment of Results

The growth rates, lag phases, and mycotoxin concentrations were evaluated by analysis of variance (ANOVA) to determine the effect of chitosan doses, a_W_, *Fusarium* species, and two- and three-way interactions. When the analysis was statistically significant, the post hoc Tukey´s multiple comparison procedure was used for separation of the means. Statistical significance was judged at the level *p* ≤ 0.001. All the analyses were done using SigmaStat for Windows Version 2.03 (SPSS Inc.)

## Figures and Tables

**Table 1:** Analysis of variance on the effects of water activity (a_W_), chitosan dose (C), different strains (S), and their interactions on growth rates of *Fusarium proliferatum* and *Fusarium verticilloides* on irradiated maize grains.

Source of Variation	df ^a^	Growth Rates	
		MS ^b^	F ^c^
S	1	0.06	1.2
C	3	0.6	14 *
a_W_	2	66.5	1364 *
S × C	3	0.2	5.6
S × a_W_	2	0.07	1.4
C × a_W_	6	1.1	22 *
S × C × a_W_	6	0.05	1.1 *

* Significant at *p* < 0.001; ^a^ degrees of freedom; ^b^ mean square; ^c^ Snedecor-F.

**Table 2:** Analysis of variance on the effects of a_W_, chitosan doses (C), and their interactions on growth rates of *Fusarium graminearum* RC22-2 and *Fusarium graminearum* RCFG6001 on irradiated wheat grains.

Source of Variation	df ^a^	Growth Rates	
		MS ^b^	F ^c^
S	1	56.4	1.8
C	3	8933.3	287.5 *
a_W_	2	7043.5	226.6 *
S × C	3	56.3	1.8
S × a_W_	2	95.9	3.1
C × a_W_	6	1095.3	35.2 *
S × C × a_W_	6	88.4	2.8

* Significant at *p* < 0.001; ^a^ degrees of freedom; ^b^ mean square; ^c^ Snedecor-F.

**Table 3:** Combined effect of different concentrations of chitosan and a_W_ on fumonisin (FB_1_ + FB_2_ + FB_3_ μg/g) accumulation by *Fusarium verticillioides* M7075 and *Fusarium proliferatum* RC2080 on irradiated maize at 25 °C.

Strain	Chitosan Dose (mg/g)	a_W_		
		0.99	0.98	0.95
*F. proliferatum* RC2080	0	2017 ± 638 ^a^	5423 ± 2028 ^a^	819 ± 364 ^a^
	0.5	2747 ± 651 ^a^	963 ± 118 ^b^	611 ± 489 ^a^
	1	1386 ± 333 ^b^	637 ± 98 ^c^	447 ± 284 ^b^
	2	2064 ± 537 ^a^	849 ± 403 ^b^	214 ± 219 ^c^
*F. verticillioides* M7075	0	4568 ± 645 ^a^	8582 ± 825 ^a^	211 ± 181 ^a^
	0.5	1532 ± 1311 ^a^	2745 ± 525 ^b^	409 ± 81 ^a^
	1	9306 ± 1487 ^b^	1481 ± 843 ^b^	343 ± 44 ^a^
	2	3653 ± 931 ^a^	442 ± 57 ^d^	525 ± 71 ^a^

Mean values of fumonisin concentration based on triplicate data with letters in common within a column and for each strain are not significantly different according to the Tukey HSD test (*p* > 0.001).

**Table 4:** Analysis of variance on the effects of water activity (a_W_), chitosan dose (C), different strains (S), and their interactions on total fumonisin (FB_1_ + FB_2_ + FB_3_) production by *Fusarium proliferatum* and *Fusarium verticillioides* strains grown on irradiated maize grains, and on deoxynivalenol production by *Fusarium graminearum* strains grown on irradiated wheat grains.

Source of Variation		Fumonisins		Deoxynivalenol	
	df ^a^	MS ^b^	F ^c^	MS ^b^	F ^c^
S	1	42,955,342.1	27.7 *	518.3	422.3 *
C	3	18,021,595.5	11.6 *	47.2	38.4 *
a_W_	2	55,800,484.1	36.0 *	43.2	35.2 *
S × C	3	5,105,970.2	3.3	27.9	22.7 *
S × a_W_	2	19,662,834.4	12.7 *	7.8	6.4
C × a_W_	6	21,210,509.7	13.7 *	12.7	10.4 *
S × C × a_W_	6	6,420,659.2	4.1	5.9	4.8 *

* Significant at *p* < 0.001; ^a^ degrees of freedom; ^b^ mean square; ^c^ Snedecor-F.

**Table 5:** Combined effect of different concentration of chitosan and a_W_ on deoxynivalenol (DON; ng/g) accumulation by *Fusarium graminearum* RCFG6001 and *Fusarium graminearum* RC22-2 on irradiated wheat at 25 °C.

Strain	Chitosan Dose (mg/g)	a_W_		
		0.995	0.99	0.98
RCFG6001	0	21001 ± 1024 ^a^	119 ± 111 ^a^	177 ± 46 ^a^
	0.5	93 ± 50 ^b^	148 ± 99 ^a^	Nd ^b^
	1	Nd ^c^	8.4 ± 6.8 ^b^	Nd ^b^
	2	Nd ^c^	Nd ^c^	Nd ^b^
RC22-2	0	32101 ± 1866 ^a^	1492 ± 489 ^a^	1918 ± 630 ^a^
	0.5	16455 ± 1723 ^b^	2951 ± 392 ^b^	339 ± 68 ^b^
	1	5892 ± 210 ^c^	4094 ± 646 ^c^	2245 ± 564 ^a^
	2	8323 ± 934 ^c^	15466 ± 1135 ^d^	393 ± 201 ^b^

Nd: not detected, lower than detection limit (<LOD). Mean values of DON concentration based on triplicate data with letters in common within a column for each strain are not significantly different according to the Tukey HSD test (*p* > 0.001).

## Supplementary Materials

The following are available online at https://www.mdpi.com/2076-0817/8/1/29/s1, Table S1: Characteristics of the Fusarium strains used.
