# Supplementary material for: Preliminary Study on the Use of Chitosan as an Eco-Friendly Alternative to Control Fusarium Growth and Mycotoxin Production on Maize and Wheat
Source: Pathogens. 2019 Mar 5;8(1):29. doi: 10.3390/pathogens8010029 (PMC6470945; doi:10.3390/pathogens8010029)
Supplement: Supplementary file 1 [file pathogens-08-00029-s001.pdf]

Table S1. Characteristics of the *Fusarium* strains used

Strain	Origin	Year	Mating population	Mycotoxin production	EF-1α (Accession n°)	EF-1α sequence
F. verticillioides M7075	Maize	1997	A	Fumonisins		AcgTCGACTCTGGCAAGTCGACCACTGTGAGTACTACCCTTG<br>ACGATGAGCTTATCGGCCATCGTAAACCCGGCCAAGACCTG<br>GCGGGGGATTTCTCAAAGAAAACATACTGATATCGCTTCACA<br>GACCGGTCACTTGATCTACCAGTGCGGTGGTATCGACAAGC<br>GAACCATCGAGAAGTTCGAGAAGGTTAGTCACTTTTCCTTCT<br>ATCGCGCGTTCTTTGCCCATCGATTCCCCCCTACGACTCGAA<br>ACGTACCCGCTACCCCGCTCGAGCCCAAAAATTTTGCGATAC<br>GACCGTAATTTTTTCTGGTGGGGCATTTACCCCGCCACTCGA<br>GCGGCGCGTTTCTGCCCTCTCCCATTCCACAACCTCACTGA<br>GCTCATCGTCACGTGTCAAGCAGTCACTAACCATCCGACAAT<br>AGGAAGCCGCTGAGCTCGGTAAGGGTTCCTTCAAGTACGCC<br>TGGGTTCTTGACAAGCTCAAGGCCGAGCGTGAGCGTGGTAT<br>CACCATCGATATCGCTCTCTGGAAGTTCGAGACTCCTCGCTA<br>CTATGTCACCGTCATTGGTATGTTGTCGCTCTTACTCCGTTCT<br>ATATCTCCTATTACTAACACATCACATAGACGCTCCCGGTCA<br>CCGTGATTTCATCAAGAACA[1]
F. proliferatum RC2080	Maize	1997	D	Fumonisins		GTCGTCATCGGCCACGTCGACTCTGGCAaGTCGACCACTGT<br>GAGTACTACCCTGGACGATGAGCTTATCTGCCATCGTGATCC<br>TGACCAAGATCTGGCGGGGTACATCTTGGAAGACAATATGCT<br>GACATCGCTTCACAGACCGGTCACTTGATCTACCAGTGCGGT<br>GGTATCGACAAGCGAACCATCGAGAAGTTCGAGAAGGTTAG<br>TCACTTTCCCTTCGATCGCGCGTCCTCTGCCCACCGATTTCA<br>CTTGCGATTCGAAACGTGCCTGCTACCCCGCTCGAGACCAA<br>AATTTTTGCGATATGACCGTAATTTTTTTGGTGGGGCATTTAC<br>CCCGCCACTCGAGCGATGAGCGCGTTTTTGCCCTTTCCTGT<br>CCACAACCTCAATGAGCGCATTGTCACGTGTCAAGCAGCGA<br>CTAACCATTCGACAATAGGAAGCCGCTGAGCTCGGTAAGGG

						TTCCTTCAAGTACGCCTGGGTTCTTGACAAGCTCAAGGCCGA GCGTGAGCGTGGTATCACCATCGATATTGCTCTCTGGAAGTT CGAGACTCCTCGCTACTATGTCACCGTCATTGGTATGTTGTC GCTCATACCTCATCCTACTTCCTCATACTAACACATCAT[2]
F. graminearum RC22-2	wheat	2002	Ability to produce homothallic perithecia	DON		TGAtgaaatCAcGgtGAcCGGGAGCGTCTGATAGCCATGTTAGTA TGAGAATGTGATGACAGCAGTGGTGACAACATACCAATGACG GTGACATAGTAGCGAGGAGTCTCGAACTTCCAGAGGGCGAT ATCAATGGTGATACCACGCTCACGCTCGGCTTTGAGCTTGTC AAGAACCCAGGCGTACTTGAAGGAACCCTTACCGAGCTCGG CGGCTTCCTATTGACAGGTGGTTAGTGACTGGTTGACACGTG ATGATGAGCGCCCAGGGAATGGTTTGTGGGAAGAGGGCAGA CGCCTGTCGCTCGAGTGGCGGGGTATGAGCCCCACCGGGG GAAAAAATTACGACAAAGCCGCAAAATTTTTGACCTCGAGCG GGGTAACAGGCGCGTATCGAGTCGTCGTGTGAGGGCGATTC GAATGATATTTCGAAAGGGAAAAGGGCGCGCGAtCGAGGAAA AtGAGACCAACCTTCTCGAACTTCTCGATGGTTCGCttGtCGAtA CCACCGCactGGtAgaTCAAGTGACcGGtcTatcaaagtatgtcagcacat tggaaatttgaaactacccc[3]
F. graminearum RCFG6001	wheat	2008	Ability to produce homothallic perithecia	DON, 15-ADON	KX359399	

[1] 100 % Ident. with *F. verticillioides* NRRL 25087, accession number: JF740717.1; [2] 100 % Ident. with *F. proliferatum* NRRL 25082, accession number: JF 740713.1; [3] 99.8 % Ident. with *F. graminearum* NRRL 40567, accession number: X087140.1
